# Health status of the old and very old people in Germany: results of the Gesundheit 65+ study

**DOI:** 10.25646/11663

**Published:** 2023-09-20

**Authors:** Beate Gaertner, Christa Scheidt-Nave, Carmen Koschollek, Judith Fuchs

**Affiliations:** Robert Koch Institute, Berlin, Germany Department of Epidemiology and Health Monitoring

**Keywords:** PUBLIC HEALTH, SURVEILLANCE, AGE, INDICATORS, HEALTH MONITORING

## Abstract

**Background:**

The demographic change makes comprehensive health reporting on health at older age an important topic.

**Methods:**

Gesundheit 65+ is a longitudinal epidemiological study on the health status of persons aged 65 and older in Germany. Based on a two-stage stratified random sample from 128 local population registers, 3,694 persons participated in the baseline survey between June 2021 and April 2022 (47.9 % women, mean age 78.8 years). Weighted prevalences for 19 indicators of the baseline survey are presented overall and by age, sex, education and region of residence.

**Results:**

Overall, 52.0 % of all participants of the baseline survey reported to be in good or very good health, and 78.5 % reported high or very high satisfaction with their life. This was in contrast to the large number of health/functional limitations whose prevalences ranged from 5.3 % for severe visual limitations to 69.2 % for multimorbidity. The health status of women was clearly worse than that of men, and the health status of persons aged 80 and older was worse than between 65 and 79 years of age. There was a clear educational gradient evident in the health status, but there were no differences between West and East Germany.

**Conclusions:**

Gesundheit 65+ provides a comprehensive database for description of the health status of old and very old people in Germany, on the basis of which recommendations for action for policy and practice can be derived.

## 1. Introduction

In recent decades, significant changes in the population structure have become apparent throughout the world: The proportion of older people is on the rise, while the proportion of younger people is decreasing. According to the Federal Statistical Office, the proportion of the total population of Germany that is of age 65 and older has risen from 15 % in 1991 to 22 % in 2021; the proportion of very old people aged 85 and older has also increased [1]. It can be presumed that the proportion of older people in society will keep increasing [2]. The current life expectancy in Germany in 2020 for women and for men is 83.2 years and 78.3 years, respectively, and will rise, albeit at a slower rate due to flu epidemics and, from March 2020, the COVID-19 pandemic [3]. Ageing is associated with a number of health challenges, including an increased likelihood of illness and a decline in physical and cognitive function. This is associated with limitations in coping with everyday life and a possible need for assistance and care [4, 5]. Accordingly, the health status of old and very old people in Germany is increasingly coming into focus. Moreover, during the COVID-19 pandemic it became clear that important information on the health status of old and very old people was not available during the pandemic [6]. Monitoring the health status of older people is therefore relevant in many respects, e.g. for planning additional health care needs, early prevention measures while ensuring equal opportunities and participation, and to future pandemic preparedness.

The ‘Global Strategy and Action Plan on Ageing and Health 2016 – 2020’ of the World Health Organisation (WHO) identified measures for political leaders worldwide that are necessary to ensure that all people have the opportunity to live a long and healthy life [7]. This was in preparation for the ‘United Nations Decade of Healthy Ageing (2021 – 2030)’ [8]. The outcome report indicated that major challenges remain to exist and that, in particular, more data on healthy ageing across the life course should be obtained. This includes information on physical and cognitive functioning and greater standardisation of measurement data [9].

In Germany, the national health goal titled ‘Healthy Ageing’ was formulated in 2012 to strengthen the physical, mental and social resources of older people, to improve the management of age-associated health problems such as multimorbidity and dementia, and to increase the quality of medical and nursing care [4, 10]. The ‘Health in Germany’ report of the Robert Koch Institute (RKI), in its chapter ‘How healthy are older people?’, highlights the major importance of recurring primary data surveys through representative sampling as a way to allow conclusions to be drawn on diseases and functional limitations and impairments in everyday life [5].

The health status at old age comprises various components that can be described through standardised measures (indicators) [11]. These indicators should be formulated appropriately such that they not only ensure a one-time description of the status, but are able to map changes over a period of time. Based on the models of the WHO and the International Classification of Functioning, Disability and Health (ICF), a concept for the classification of indicators was developed within the framework of the ‘Improving Health Monitoring in Old Age (IMOA)’ project funded by the Robert Bosch Foundation from 2016 to 2018, which includes the health areas of ‘environmental factors’, ‘activities and participation’ and ‘personal factors (i.e. health/functional resources)’. In these health areas, a set of indicators for description of the health status for the age group 65+ years was developed in a multi-stage structured consensus process, in which an interdisciplinary committee of experts was involved [12]; the following presentation is based on this set of indicators.

Gesundheit 65+ (see Study on Health of Older People in Germany (‘Gesundheit 65+’): objectives, design and implementation) [13] and its baseline survey provide a data set that can be used to represent some of the core indicators described in IMOA as well as other relevant indicators (e.g. visual, hearing and mobility impairments). This will contribute to the description of the health status of older people in Germany at the time of the COVID-19 pandemic, including their health resources and risks.

**The Gesundheit 65+ Study**

Extension of the ongoing RKI monitoring by including the old and very old people with functional impairments.

**Data holder:** Robert Koch Institute

**Objectives:** Core component for a comprehensive public health monitoring in the 65+ population with a focus on subjective, psychosocial, functional aspects of health and closure of existing data gaps related to the health and well-being of older people during and after the COVID-19 pandemic.

**Study design:** Longitudinal survey (baseline survey plus three follow-ups every four months; home visit examination parallel to the last follow-up).

**Statistical population:** German-speaking persons aged 65 and older who live in Germany and are registered there as their main place of residence

**Sampling:** Two-stage sampling procedure: 1) 128 randomly selected municipalities and cities nationwide, 2) stratified random sampling in the respective local population registers according to gender and two age groups (65 – 79, 80 + years of age).

**Survey modes (mixed-mode):** Paper-based or online questionnaire, interview by telephone or face-to-face

**Proxy participation:** Permitted

**Consent by a legal representative:** Permitted

**Sample size:** 3,694 participants, of which 2,175 were 80 years of age and older

**Total data collection period:** June 2021 to April 2023

**Data collection period of the baseline survey:** June 2021 to April 2022

For more information, please visit www.rki.de/gesundheit65plus

## 2. Methods

### 2.1 Study design and sampling

As part of the RKI’s health monitoring programme, the nationwide, population-based, longitudinal epidemiological study titled Gesundheit 65+ (Health 65+) was conducted between June 2021 and April 2023 to collect representative data on the health status of old and very old people in Germany during the COVID-19 pandemic (Infobox). The target population comprised permanent residents of Germany aged 65 and older. People with insufficient knowledge of the German language and people who had died or moved away before the start of the study or were untraceable were excluded from the study. Participation was thus possible regardless of the health status of the invited person, e.g. persons in nursing homes or with limited capacity to provide consent were included. In order to also include old and very old people with functional limitations in the study, a study design that was previously tested for this age group was used for contacting and data collection [14, 15]. The baseline survey of the study was conducted using a mixed-mode survey design of data collection modes (paper/online questionnaire, interview on the phone or during a home visit) between June 2021 and April 2022. In order to reduce barriers to participation in the study, assistance in participating by relatives or other close persons or proxy-participation was permitted. For a detailed description of the study including the longitudinal data collection with three follow-ups and a home visit examination over a period of 12 months after baseline, see elsewhere [13].

The sampling was conducted in a two-stage, stratified cluster sampling procedure. In the first stage, 128 primary sampling units (PSUs) were drawn at random from all municipalities in Germany. In the second stage, within the PSUs, sex- and age-stratified random samples of the population in the age groups 65 to 79 years and 80 years and older were then drawn from the local registration registers. The contacting of the drawn individuals for the baseline survey and thus for study participation was done according to the previously developed and tested sequential mixed-mode design [14]: written, telephone and face-to-face. Since non-contacts were visited on site in the last step of contacting and this was not feasible in terms of personnel in all PSUs at the same time, the 128 PSUs were randomly assigned into 32 routes with four PSUs each. These routes were then scheduled in an approx. 9-month route plan and persons were invited to participate in the study successively according to this plan. However, due to the pandemic in November 2021, face-to-face contacting and interviews had to be discontinued. A total of 12,248 people were invited to participate in Gesundheit 65+, and 7,904 of the invited persons were 80 years of age or older. Of those invited, 307 had to be excluded from participation in the study for the reasons mentioned above. According to the ‘Standards of the American Association for Public Opinion Research’ [16], the response rate 2 (i.e. including partial surveys) was 30.9 % as a total of 3,694 persons participated in the baseline survey. The majority of the participants participated by paper-based questionnaires (86.2 %) followed by online questionnaires (7.5 %), face-to-face interviews (4.1 %) and telephone (2.2 %) interviews. In total, there were 327 proxy-participations.

### 2.2 Indicators

The content of the baseline survey included essential health concepts for old and very old people from the health areas of environmental factors, activities/participation and health/functional resources [17]. The selection of indicators was based, to the extent possible, on the health indicators for the population aged 65 and older previously developed in IMOA [12] and supplemented to include other important topics related to the health of older people (e.g. visual, hearing and mobility impairments). In order to lower the barriers to participation for very old or functionally impaired persons, the effort involved in responding in the survey was minimised as far as possible and, e.g. extensive instruments were not administered.

#### Environmental factors

Receiving long-term care benefits (Pflegegrad) was recorded by asking ‘Do you have a degree of care?’, and the responses were summarised in two categories (yes vs. no or application is pending). Regarding the provision of care to another person in the form of informal lay care, the participants were asked whether they ‘currently took care of or looked after a person in need of care or who is seriously ill’ (yes vs. no). Social support was measured with the Oslo-3 Social Support Scale [OSS-3, 18], a three-question instrument assessing the number of close persons, concern and interest of other people, and receiving practical help from neighbours (range of the total score: 3 to 14). A total score of less than 9 was considered as low level of support (yes vs. no) [19]. Loneliness was assessed with the three-question Revised UCLA Loneliness Scale instrument [R-UCLA, 20, 21], which queried lacking companionship, feeling left out and feeling isolated from others (range of total score: 3 to 9). Loneliness was defined by a total score of 6 or more (yes vs. no) [22].

#### Activities/participation

Limitations in activities of daily living were assessed through internationally established instruments of the European Health Interview Survey (EHIS) [23]. Five basic activities of daily living (intake of food, getting up or sitting down, dressing and undressing, using toilets, personal hygiene) were recorded following Katz et al. [24]. In addition, seven instrumental activities of daily living (preparing meals, using the telephone, going shopping, organising medication intake, doing light housework, doing occasional heavy housework, taking care of finances and everyday administrative tasks) were used following Lawton and Brody [25]. For each activity, the participants were asked whether they would normally have difficulty performing that activity without assistance (response categories: no, some, a lot of difficulty, unable to do; additional category for instrumental activities only: not applicable (never tried or done this). If a lot of difficulty or impossibility to carry out the activity was reported at least once, this was defined to be a limitation in basic or instrumental activities of daily living (yes vs. no). The additional response category of ‘not applicable’ was not counted as a limitation, and two missing values each were permitted.

#### Health/functional resources

The self-perceived general health was recorded by asking ‘How is your health in general?’ (response categories: very good, good, fair, bad, very bad) [26, 27]. For the analyses, the response categories ‘good’ and ‘very good’ were combined and compared to the other three categories. Self-reported 12-month prevalences of ten different age-relevant chronic diseases and health problems were recorded based on a list according to the EHIS [23]. These diseases and health problems comprised 1. Hypertension (high blood pressure), 2. Coronary heart disease (incl. myocardial infarction or chronic symptoms secondary to myocardial infarction, angina pectoris), 3. Stroke (incl. chronic symptoms secondary to a stroke), 4. Hypercholesterolaemia (high blood lipids), 5. Diabetes, 6. Chronic bronchitis (incl. chronic obstructive pulmonary disease, emphysema), 7. Arthrosis, 8. Osteoporosis, 9. Lower back disorder or other chronic back defect, and 10. Depression. In addition, cancer was recorded by asking ‘Has a doctor ever diagnosed you with cancer?’ First, the total sum of the prevalent diseases and health problems was calculated from the responses given (range: 0 to 11). Up to seven missing values were permitted, as it was assumed that in the selected list format, diseases which the participants were not afflicted by or which were unknown to them often remained without a response. Multimorbidity was defined as the presence of two or more diseases and health problems (yes vs. no) [28]. Depressive symptoms in the past two weeks were assessed with the two-question Patient Health Questionnaire (PHQ-2) instrument [29] on symptoms of little interest or pleasure in doing things, as well as feeling down, depressed or hopeless (range of total score: 0 to 6). A total score of 3 or more was considered to indicate depressive symptoms (yes vs. no). General satisfaction with life was assessed by asking ‘How satisfied are you, all things considered, with your life at present?’ (response categories: 0 – completely dissatisfied to 10 – completely satisfied) following Richter [30]. Any score of 7 or more was considered to indicate high or very high satisfaction with life [12].

Sensory and mobility limitations were recorded according to the EHIS [23] using five questions: one question on vision, two questions on hearing and two questions on mobility. The response categories were identical for all questions (no, some, a lot of difficulty, unable to do). If participants reported at least a lot of difficulty in their vision even with glasses or contact lenses, this was defined as severe visual impairment. The coding for severe hearing or mobility impairments was done accordingly. Persons who reported at least a lot of difficulty hearing a conversation with another person (with a hearing aid, if applicable) in either a quiet (1st question) or noisier room (2nd question) were coded as experiencing severe hearing impairment. Mobility impairment was considered if the person reported difficulty walking half a kilometre on level ground without a walking aid (1st question) or walking up or down 12 steps (2nd question). For all other responses or response combinations, no impairments were assumed.

Pain was recorded by asking about the intensity of pain during the past four weeks (response categories: none, very mild, mild, moderate, severe, very severe) [31]. Participants reporting pain were asked how long the pain had been persisting [32]. Chronic pain was assumed if severe or very severe pain for at least six months was reported. Falls were recorded according to the recommendations of the PROFANE network [33] by asking ‘Have you fallen, tripped or slipped so that you lost your balance and landed on the ground or a lower level during the past 12 months?’. In the analyses, the next question on the number of falls was used to construct two variables, i.e. whether the participant had fallen at least once or at least twice (yes vs. no in each case). Urinary incontinence in the past 12 months (yes vs. no) was evident if participants reported urinary incontinence or problems controlling their bladder [23]. Faecal incontinence was recorded by asking ‘Have you had difficulty holding or controlling your bowel movements in the past four weeks? (yes vs. no). Subjective memory impairment was evident if the participants reported worsening of their memory and were worried about it [34].

### 2.3 Stratification variables

Information on gender, month and year of birth was provided from the registration data by the local population registers at the time of sampling. With regard to gender, the provision of a third gender was permitted, but no one used this option, so that a stratification for women and men was done in the analyses. Age in years was calculated using the date of birth and the day of participation in the survey. Two age groups were defined (65 to 79 vs. 80 years and older). There are several established classifications for determining the level of education [vgl. 35]. For the following analyses, the Comparative Analyses of Social Mobility in Industrial Nations (CASMIN) classification [36, 37] was chosen to represent the educational level, as this classification maps the specifics of the German tripartite school system quite well for the analyses and if applicable may better identify social inequalities. The CASMIN classification distinguishes between low education (i.e. primary and low secondary education), medium education (i.e. intermediate/high secondary education) and high education (i.e. tertiary education) on the basis of the highest level of school and vocational/professional education attained [vgl. auch 38]. In order to map regional differences, the region of residence variable distinguished between the federal states of West and East (incl. Berlin) Germany.

### 2.4 Statistical analysis

First, absolute numbers and percentages were calculated for a sample description overall and by gender. Then, prevalences and 95 % confidence intervals (95 % Cl) of all health indicators were calculated overall and by the specified stratification variables and presented in a table or figure. Prevalences are estimates of the percentage of affected persons in the target group at a given time. Their precision can be assessed using confidence intervals – broad confidence intervals indicate greater statistical uncertainty of the results. Since subjective assessments were required for some indicators (i.e. loneliness, depressive symptoms, satisfaction with life and subjective memory impairment), only self-reported data from the invited individual and no data from proxies were taken into account for these indicators in the analyses.

A weighting factor was calculated in order to correct the prevalences for deviations of the study participants from the target population of people aged 65 and older in Germany as of 31st December 2020 with regard to gender, age, region and municipality size according to the BIK-10 classification [39]. In addition, the weighting factor took into account deviations in the level of education compared to the resident population of Germany based on the 2018 Microcensus according to the International Standard Classification of Education (ISCED classification) [40].

All analyses were conducted using Stata/SE 17.0 (Stata Corp., College Station, TX, USA, 2017). In order to appropriately account for clustering by PSUs and the weighting, all analyses were conducted using survey procedures. A statistically significant difference between groups is assumed to exist when confidence intervals do not overlap. All differences that are significant according to this definition are reported in the results section.

## 3. Results

### 3.1 Description of the baseline sample

Of the 3,694 participants in the baseline survey of Gesundheit 65+, 47.9 % were women and 58.9 % were 80 years of age or older (Table 1). On average, the participants were 78.8 years of age and the maximum age was 100 years. A total of 49,2 % of all participants had a low educational level and 23,5 % a high educational level. A total of 19.3 % of the participants resided in East Germany.

There was a difference between women and men with regard to education: Women were more often assigned to a low level of education (54.5 % vs. 44.3 %) than men, and less often to a high level of education (15.0 % vs. 31.4 %) than men.

### 3.2 Health of older people in Germany according to the different health areas

The prevalences and the number of missing values of the selected health indicators are shown in Table 2 for the health areas of environmental factors, activities/participation and health/functional resources. Accordingly, the absolute number of missing values for the indicators for the 3,694 participants varied from 52 missing values on self-perceived general health to a maximum of 223 missing values on receiving long-term care benefits; i.e. the percentage of missing values ranged between 1.4 % and 6.0 %.

In the description of their environmental factors, 16.9 % of the older persons reported that they received long-term care benefits. A total of 11.8 % of the participants provided informal care to a person in need of care or who was seriously ill. Overall, 19.2 % of the older people received a low level of social support and 19.2 % felt lonely. In the field of activities/participation, 9.8 % and 20.3 % of the older people were limited in basic and instrumental activities of daily living, respectively. Concerning their health resources, 52.0 % of the older persons assessed their health as good or very good, and 78.5 % were satisfied or very satisfied with their lives. However, health and functional limitations were quite common: Multimorbidity (69.2 %), urinary incontinence (27.5 %), subjective memory impairment (27.3 %), at least one fall in the past year (24.1 %), impaired mobility (20.8 %), severe hearing impairment (17.0 %), chronic pain (14.3 %), depressive symptoms (13.8 %), multiple falls in the past year (12.9 %), faecal incontinence (9.6 %) and severe visual impairment (5.3 %).

### 3.3 Gender and age differences in different health status areas

**Figure 1:**
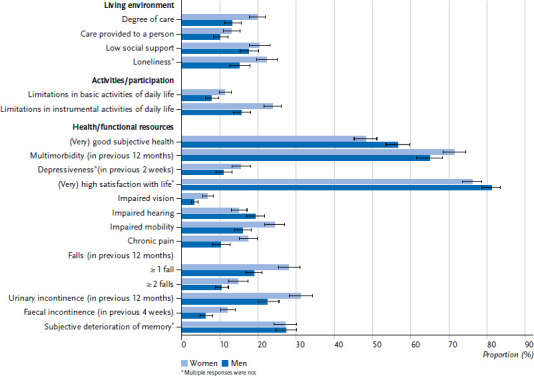
Prevalences and 95 % confidence intervals of health indicators by gender (n = 3,694, weighted analyses, numbers are in %) Source: Gesundheit 65+, own description

Overall, women aged 65 and older rated their health worse than men (Figure 1). Accordingly, they reported urinary incontinence (31.4 % vs. 22.6 %), at least one fall in the past year (28.2 % vs. 19.0 %), impaired mobility (24.4 % vs. 16.1 %), limitations in instrumental activities of daily living (23.9 % vs. 15.8 %), loneliness (22.3 % vs. 15.2 %), receiving long-term care benefits (19.9 % vs. 13.3 %), chronic pain (17.4 % vs. 10.3 %), depressive symptoms (15.6 % vs. 11.1 %), multiple falls in the past year (14.8 % vs. 10.5 %), faecal incontinence (12.1 % vs. 6.4 %), limitations in basic activities of daily living (11.4 % vs. 7.8 %) and severe visual impairments (6.9 % vs. 3.4 %) more often than men. Correspondingly, men reported good or very good subjective health more often than women (56.8 % vs. 48.3 %).

Table 3 provides an overview of the health status stratified by gender and age groups. The health status of very old persons aged 80 and older was clearly worse compared to the younger age group of 65 to 79 years of age (Table 3). In some cases, the prevalences were several times higher in the older age group than in the younger age group (e.g. 6.1 % of the 65- to 79-year-old and 44.3 % of the 80-year-old and older women received long-term care benefits, respectively). This applied equally to both women and men with regard to receiving long-term care benefits, limitations in basic and instrumental activities of daily living, subjective health status, limitations in sensory and mobility, chronic pain, fall events and urinary and faecal incontinence. With regard to depressive symptoms and lower life satisfaction, this only applied to men, and with regard to loneliness and multimorbidity, only to women. Women of very old age were also less likely to care for another person informally than younger women.

### 3.4 Educational differences in different health areas

There was a clear educational gradient in the health status in old age (Table 4). Older women and men with a low level of education, and to some extent also with a medium educational level, reported health problems more frequently than those with a high educational level. Occasionally, the prevalences were several times higher among those with a low educational level than with a high educational level (e.g. 6.7 % of the women with a high educational level and 25.9 % of the women with a low educational level received long-term care benefits, respectively). This applied equally to both women and men with regard to receiving long-term care benefits, limitations in basic and instrumental activities of daily living, subjective health status, severe visual and mobility impairments. With regard to loneliness, depressive symptoms, lower life satisfaction and urinary incontinence, an educational gradient was only detected for men, whereas the same applied to low social support and chronic pain only for women.

### 3.5 Living in West vs. East Germany and health of older persons in different health areas

No regional differences in the health of older persons by residence in West or East Germany were detected (Table 5). The prevalences of the selected health indicators differed between West and East Germany by a maximum of 3.5 %, i.e. in the case of subjective health status. None of the differences was statistically significant.

## 4. Discussion

Gesundheit 65+ is the first nationwide health survey including an examination of the population aged 65 and older in Germany, with special consideration of the very old people and persons with health impairments. The present analyses sheds light on the health status of older women and men in Germany during the COVID-19 pandemic from June 2021 to April 2022 on the basis of the population-representative baseline survey. Overall, 78.5 % of the participants were very satisfied with their lives and every second woman and man assessed their own health as good or very good. This contrasts with a large number of health limitations being reported; the phenomenon is described in the literature as a ‘well-being paradox’ and is presumably based on adaptation strategies of the older people [41]. However, with regard to subjective health status as well as health impairments and receiving long-term care benefits, there are considerable gender differences and according to education, which become even more pronounced beyond the age of 80. Our data revealed a noticeably higher prevalence of receiving long-term care benefits compared to the presence of limitations in basic activities of daily living (16.9 % vs. 9.8 %). This can be explained by the fact that more and different criteria (e.g. communication skills, mental disorders, cognitive impairments) are used in the determination of long-term care benefits. Furthermore, no regional differences between West and East Germany were detected in Gesundheit 65+.

The age- and gender-specific differences in health status described are essentially consistent with those from other national and international studies. Overall, women rate their health as worse and report more health problems than men, but they live longer. These differences, also known as the gender paradox, have been known for many years [42–46] and can also be found in recent studies [47–51]. The underlying reasons are varied and complex, and are attributed to both biological (sex) and social (gender) factors, with no underlying monocausal relationship [52]. Comparing the participants of Gesundheit 65+ it was evident that women were more likely to be less educated than men. The gender differences detected are thus partly due to the unequal social status, as has been discussed since the mid-1990s [53–55].

Health inequalities in old age in Germany have been described previously by other authors [56–59]. Gesundheit 65+ also shows health inequalities by educational level for older age in Germany, some of which pertain to both sexes (receiving long-term care benefits, self-rated general health, limitations in activities of daily living, impairments of vision and mobility), some only to women (low social support, chronic pain) or only to men (loneliness, depressive symptoms, low satisfaction with life, urinary incontinence). Participants with a high level of education had fewer health problems than those with a low level of education. The extent to which these differences pre-existed in the present sample before reaching older age and are persisting, increasing or decreasing in this phase of life cannot be clarified with this cross-sectional analysis of the baseline survey of Gesundheit 65+. In addition to longitudinal analyses to address this issue, it would also be necessary in the future to consider other aspects of the social status, such as poverty risk, which pertains to women in particular [60], and other indicators of social inequality in further analyses.

In the following, selected results are discussed that provide a deeper insight into the lives of older and very old people, especially during the COVID-19 pandemic, and thus indicate which topics should be given greater attention, also with regard to future pandemics. Since Gesundheit 65+ was conducted only during the time of the COVID-19 pandemic and no pre-pandemic data on participants being available, it is not possible to assess the effects of the pandemic on the health status of older people in Germany.

Psychosocial health was an important topic during the COVID-19 pandemic. At the beginning of the pandemic, older people were in focus as a high-risk group, not only in Germany; especially with regard to negative indirect health consequences of the containment measures (distancing, contact reduction, discontinuation of social activities) such as social isolation, loneliness, lack of social support and a deterioration of mental health [6]. Subsequent research was able to corroborate this in part [61], though younger age groups were mainly afflicted. During the survey period of Gesundheit 65+, the COVID-19 pandemic containment measures were again intensified from the end of 2021 due to the high risk of infection by the omicron variant of SARS-CoV-2. Against this background, a total of one in five people in Gesundheit 65+ described themselves to be lonely. Loneliness was more pronounced in women than in men, which is consistent with a Japanese study conducted during the same time period [50]. The present results can be compared only to a limited extent to the results from the German Ageing Survey (DEAS) 2020/21, which are also presented in this issue of the Journal of Health Monitoring, which is due to methodological reasons: a different instrument was used, persons over 90 years of age were excluded from the study and the data were collected in a telephone interview. With regard to social support, it became clear in Gesundheit 65+ that the majority of participants receive social support, though to a decreasing degree with increasing age, which is consistent with other studies [62]. The effects of social isolation and loneliness on the health of older people are well known [63, 64], e.g. as a risk factor for dementia [65]. This aspect can be considered further in the future in longitudinal analyses of Gesundheit 65+, e.g. to analyse the correlation between loneliness and morbidity or mental health in more detail. Women were particularly likely to be afflicted by depressive symptoms in Gesundheit 65+; the same applied to men 80 years of age and older or with low education levels.

With regard to health limitations, the results from Gesundheit 65+ show that multimorbidity is a common phenomenon among older people, which is consistent with other studies [66, 67], and that the prevalence varies according to age and gender [68, 69]. People with multimorbidity are more likely to be admitted to hospital, to be prescribed more medication and to have a higher risk of mortality [67, 68, 70]. The bidirectional correlation between multimorbidity and functional limitations that limit activities and participation is well known [71]. These include severe impairments in vision, hearing, cognition, and associated limitations in activities of daily living. In line with other studies, these were reported more frequently with increasing age [72–74]. No gender, age or educational differences were detected for subjective memory impairment. The fact that more than a quarter of the population aged 65+ report memory impairment indicates that there is a need for health care and, if necessary, diagnostics, as subjective memory impairment is considered to be a risk factor for cognition decline, future dementia and mortality [75–78].

Over a quarter of the participants of Gesundheit 65+ report urinary incontinence and the respective proportion increases significantly with age, as has been reported in other studies as well [79–81]. Women are more likely to report urinary and faecal incontinence than men. As these are shameful issues with serious effects on the quality of life and participation of those afflicted, they should be regularly addressed in medical consultations in accordance with existing guidelines [82] and preventive measures such as pelvic floor training should be offered at an early stage. Incontinence is also associated with frailty [83]. At least one fall event in the past year was common especially among the participants of Gesundheit 65+ aged 80 years and older, with over 30 % among men and over 40 % among women, a similar prevalence as described by the WHO as early as in 2007 [84]. Falls result in moderate to severe injuries, hospitalisation, fear of falling, loss of independence and premature death [85, 86], which can be prevented or reduced by adequate intervention programmes (personal, medication and environmental measures), but there is still a need to catch up in the implementation of such measures [87].

A comparison of the results with other national ageing studies is only possible to a limited extent due to the different methodological approaches. For example, both the DEAS [88] and the Corona Survey of the Survey of Health, Age and Retirement (SHARE) [89] only collect data from previous panel participants in their 2020/21 wave due to the pandemic. The D80 + study, on the other hand, only surveyed people aged 80 and older with a focus on life situation and quality of life and used other instruments or operationalisations (e.g. for multimorbidity) [90]. For comparison of Gesundheit 65+ and the telephone survey of private households GEDA 2019/2020-EHIS [91], separate further methodological studies are required before a classification of deviating prevalences is possible, e.g. with regard to the prevalence of informal care [92].

###  

#### Strengths and limitations

Gesundheit 65+ delivers representative data on the health status and related factors for the older and very old population in Germany. The study included both persons in private households as well as in institutions, without and with severe limitations in health, and proxy participation as well as consent by legal representatives were permitted. The mixed-mode survey design, which allowed for participation via a paper-based, web-based, telephone or face-to-face questionnaire/interview, made it possible for persons to participate who are usually excluded or do not participate in other studies (e.g. persons with severe visual or hearing impairments, support needs or lack of capacity to consent). This is the particular strength of Gesundheit 65+. Due to these measures and the elaborate recruitment process, a good response rate and sample composition could be attained for this age group [93]. For example, there is a good agreement in the proportion of Gesundheit 65+ participants who receive long-term care benefits (17 %) compared to 19 % among those 65 years of age or older in the general population of Germany according to the data of the Federal Statistical Office [94] (own calculations).

The present study has some limitations as well. The aim of Gesundheit 65+ was to attain a sample that was equally distributed by gender by applying stratified random sampling. However, the proportion of women of 47.9 % is lower than that of men. The willingness of invited men to participate was thus higher than that of women, which was balanced out by weighting in the analyses. Further response analyses in Gesundheit 65+ should address this issue in the future, e.g. the inclusion of people in nursing homes and socially disadvantaged neighbourhoods. Gesundheit 65+ will not be able to close the existing data gap especially with regard to the status of older persons in nursing homes, as analyses from the previous studies OMAHA I and the IMOA feasibility study show [95, 96]. Other methodological approaches and monitoring systems are therefore needed for health monitoring in nursing homes [97]. Persons with insufficient knowledge of the German language were excluded from the study, as it was not possible to offer this group adequate participation (e.g. by means of translated questionnaires) in the context of Gesundheit 65+, as was done in other studies by the RKI [98]. In the future, it will be important to expand and link the approaches of Gesundheit 65+ to this population group as well, in order to be able to draw conclusions concerning the health status of all older persons with a migration history, regardless of their knowledge of German.

To minimise the survey participation burden, short survey instruments (e.g. the PHQ-2 instead of the PHQ-8/ PHQ-9) or single questions per topic were often used (e.g. on satisfaction with life). The use of instruments or questions that are as simple as possible should enable people with limited reading skills or cognitive function to respond. Accordingly, Gesundheit 65+ allows to paint a broad picture of the health status of older persons in Germany. On the other hand, in-depth statements or analyses on a topic are possible to a limited degree only. For example, on the topic of pain, no information can be given on the location of pain or current treatment. Due to the follow-ups over a period of one year, the home visit examination and the integration of external data sources, Gesundheit 65+ will in future offer a wide range of options for analysis of the health status and resources and risk factors of those aged 65 and older in Germany. This also applies to other relevant concepts of healthy ageing that could not be adequately represented by the baseline survey. For example, only in the future it will be possible to analyse the age-relevant concept of frailty [99] with additional measurement data from the home visit examination, e.g. on hand grip strength and cognitive function.

#### Outlook

Gesundheit 65+ presents a comprehensive database for description of the health status of old and very old people in Germany. It comprises a) survey data from four waves over the course of one year, b) examination data parallel to the last health questionnaire/interview and c) linkage to external data sources [see also 13]. The latter includes data from the statutory health insurance funds, an all-cause mortality follow-up over 20 years via the residents’ registration offices (i.e. a query on vital status and, if applicable, the notification of the date of death) and data describing the living environment via geographic information systems [100].

In the future, this database can be used, for example, to describe longitudinal associations between physical and cognitive functioning, depressive symptoms and mortality risks. The objective measures of the examination can be used to assess the consistency with self-reports (e.g. on subjective memory impairment or on height and weight) in future analyses. Gesundheit 65+ contributes to the description of resources and risk factors related to the health status of older and very old. Based on this, recommendations for action for policy and practice can be derived.

## Key statement

- Gesundheit 65+ is a study on the health status of old and very old people in Germany, and also includes functionally impaired older people.
- A total of 78.5 % of people aged 65 and older in Germany are very satisfied with their lives and every second person rates their own health as good or very good.
- Older people are afflicted by a variety of health problems and limitations.
- Especially women and persons aged 80 and older or those with low educational level report health problems and limitations particularly frequently.
- There are no regional differences evident in the health status of the older people in a comparison of West and East Germany.

## Figures and Tables

**Table 1:** Sample description overall and by gender (n = 3,694, unweighted analyses) Source: Gesundheit 65+, own description

Total (n = 3,694)		Women (n = 1,771)	Men (n = 1,923)
**Age group – % (n)**			
65 – 79 years	41.1 (1,519)	40.8(722)	41.5(797)
80 + years	58.9 (2,175)	59.2 (1,049)	58.6 (1,126)
**Age (years)**			
Mean (standard deviation)	78.8 (7,5)	79.0 (7,8)	78.6 (7,2)
Range	65–100	65–100	65–100
**Educational group – % (n)**			
Low	49.2 (1,793)	54.5(954)	44.3(839)
Median	27.3(994)	30.5(534)	24.3(460)
High	23.5(858)	15.0(263)	31.4(595)
**Region of residence – % (n)**			
East Germany	19.3(714)	19.5(345)	19.2(369)
West Germany	80.7 (2,980)	80.5 (1,426)	80.8 (1,554)

**Table 2:** Prevalences of health indicators overall and absolute number of their missing values (n = 3,694, weighted analyses) Source: Gesundheit 65+, own description

%		(95 % Cl)	Number missing values
**Living environment**			
Degree of care	16.9	(15.3 – 18.7)	223
Care provided to a person	11.8	(10.3 – 13.4)	74
Low social support	19.2	(17.3 – 21.3)	204
Loneliness^*^	19.2	(17.3 – 21.2)	77
**Activities/participation**			
Limitations in basic activities of daily life	9.8	(8.6 – 11.2)	82
Limitations in instrumental activities of daily life	20.3	(18.6 – 22.2)	67
**Health/functional resources**			
(Very) good subjective health	52.0	(49.6 – 54.4)	52
Multimorbidity (in previous 12 months)	69.2	(66.9 – 71.5)	140
Depressiveness^*^ (in previous 2 weeks)	13.5	(12.1 – 15.2)	89
(Very) high satisfaction with life^*^	78.5	(76.5 – 80.4)	90
Severely impaired vision	5.3	(4.6 – 6.3)	91
Severely impaired hearing	17.0	(15.5 – 18.5)	161
Impaired mobility	20.8	(18.9 – 22.8)	64
Chronic pain	14.3	(12.7 – 16.0)	143
Falls (in previous 12 months)			
≥ 1 fall	24.1	(22.3 – 26.1)	67
≥ 2 falls	12.9	(11.4 – 14.5)	103
Urinary incontinence (in previous 12 months)	27.5	(25.4 – 29.8)	60
Faecal incontinence (in previous 4 weeks)	9.6	(8.3 – 11.0)	60
Subjective deterioration of memory^*^	27.3	(25.1 – 29.7)	132

CI = confidence interval

^*^ = no proxy information included

**Table 3:** Prevalences of health indicators by gender and age (n = 3,694, weighted analyses) Source: Gesundheit 65+, own description

Women					Men			
Age group	65 – 79 years (n = 722)		80 + years (n = 1,049)		65 – 79 years (n = 797)		80 + years (n = 1,126)	
	%	(95 % Cl)	%	(95 % Cl)	%	(95 % Cl)	%	(95 % Cl)
**Living environment**									
Degree of care	6.1	(4.3 – 8.7)	44.3	(40.1 – 48.6)	8.0	(5.7 – 11.1)	26.9	(23.2 – 30.9)
Care provided to a person	16.0	(13.0 – 19.6)	7.7	(5.9 – 10.0)	10.9	(8.5 – 14.0)	8.2	(6.5 – 10.3)
Low social support	18.4	(15.1 – 22.3)	24.1	(20.6 – 27.9)	17.6	(14.5 – 21.1)	18.1	(15.4 – 21.2)
Loneliness^*^	19.4	(16.0 – 23.3)	29.0	(25.1 – 33.2)	14.4	(11.5 – 17.9)	17.5	(14.6 – 20.8)
**Activities/participation**									
Limitations in basic activities of daily life	2.9	(1.8 – 4.7)	27.0	(23.3 – 31.1)	4.9	(3.2 – 7.5)	15.3	(12.7 – 18.3)
Limitations in instrumental activities of daily life	10.1	(7.7 – 13.3)	48.7	(44.1 – 53.2)	10.4	(8.0 – 13.5)	29.7	(25.5 – 34.3)
**Health/functional resources**									
(Very) good subjective health	57.8	(53.5 – 62.0)	31.0	(27.6 – 34.7)	62.5	(58.4 – 66.4)	42.4	(39.0 – 45.8)
Multimorbidity (in previous 12 months)	66.2	(61.9 – 70.3)	82.4	(79.1 – 85.3)	63.6	(59.1 – 67.9)	70.9	(67.4 – 74.1)
Depressiveness^*^ (in previous 2 weeks)	13.8	(11.0 – 17.3)	19.6	(16.3 – 23.3)	9.4	(7.1 – 12.3)	15.8	(12.7 – 19.4)
(Very) high satisfaction with life^*^	78.2	(74.5 – 81.4)	71.9	(67.6 – 75.8)	83.8	(80.1 – 86.8)	74.4	(70.5 – 78.1)
Severely impaired vision	2.6	(1.5 – 4.5)	14.6	(12.0 – 17.7)	1.8	(1.0 – 3.1)	7.5	(5.6 – 10.1)
Severely impaired hearing	7.1	(5.1 – 10.0)	29.4	(25.6 – 33.6)	15.2	(12.5 – 18.4)	30.0	(26.6 – 33.6)
Impaired mobility	10.3	(7.7 – 13.7)	49.9	(45.4 – 54.4)	10.1	(7.9 – 13.0)	31.2	(27.7 – 35.1)
Chronic pain	14.8	(11.8 – 18.4)	22.2	(19.2 – 25.6)	8.4	(5.9 – 11.9)	14.9	(12.1 – 18.2)
Falls (in previous 12 months)								
≥ 1 fall	21.0	(17.6 – 24.8)	41.2	(36.9 – 45.7)	14.0	(11.4 – 16.9)	31.8	(28.7 – 35.1)
≥ 2 falls	8.6	(6.2 – 11.8)	26.0	(21.9 – 30.5)	7.2	(5.3 – 9.8)	19.0	(16.3 – 22.0)
Urinary incontinence (in previous 12 months)	21.9	(18.1 – 26.3)	48.4	(43.9 – 52.9)	18.0	(15.0 – 21.5)	34.5	(30.7 – 38.4)
Faecal incontinence (in previous 4 weeks)	7.9	(5.7 – 10.7)	19.7	(16.7 – 23.1)	3.8	(2.5 – 5.8)	12.9	(10.4 – 15.8)
Subjective deterioration of memory^*^	25.0	(21.6 – 28.8)	32.2	(28.0 – 36.6)	25.6	(22.3 – 29.1)	32.7	(29.0 – 36.6)

CI = confidence interval

^*^ = no proxy information included

**Table 4:** Prevalences of health indicators by gender and education (n = 3,645, weighted analyses) Source: Gesundheit 65+, own description

Women							Men					
Educational group^♦^	Low (n = 954)		Middle (n = 534)		High (n = 263)		Low (n = 839)		Middle (n = 460)		High (n = 595)	
	%	(95 % Cl)	%	(95 % Cl)	%	(95 % Cl)	%	(95 % Cl)	%	(95 % Cl)	%	(95 % Cl)
**Living environment**												
Degree of care	25.9	(22.8 – 29.3)	12.7	(9.7 – 16.5)	6.7	(4.0 – 10.9)	18.1	(14.5 – 22.3)	11.6	(8.2 – 16.1)	4.8	(3.4 – 6.9)
Care provided to a person	12.8	(10.0 – 16.2)	13.5	(9.9 – 18.1)	12.5	(8.2 – 18.6)	8.0	(5.8 – 10.9)	12.6	(8.5 – 18.3)	9.9	(7.1 – 13.6)
Low social support	23.4	(19.5 – 27.8)	16.8	(12.8 – 21.7)	12.2	(7.8 – 18.7)	19.5	(15.6 – 24.0)	18.7	(14.3 – 24.1)	12.7	(9.6 – 16.7)
Loneliness^*^	22.8	(19.3 – 26.8)	22.4	(18.0 – 27.6)	18.4	(12.7 – 25.9)	15.2	(11.7 – 19.6)	18.4	(14.1 – 23.7)	9.8	(7.4 – 12.9)
**Activities/participation**												
Limitations in basic activities of daily life	14.9	(12.4 – 17.7)	7.3	(5.3 – 10.0)	3.2	(1.6 – 6.2)	11.0	(8.1 – 14.8)	6.8	(4.5 – 10.2)	2.4	(1.3 – 4.3)
Limitations in instrumental activities of daily life	29.7	(26.5 – 33.1)	16.5	(12.8 – 21.0)	10.5	(7.3 – 15.0)	21.8	(17.9 – 26.2)	13.0	(9.7 – 17.2)	6.8	(4.8 – 9.4)
**Health/functional resources**												
(Very) good subjective health	43.7	(39.3 – 48.2)	52.4	(46.7 – 58.0)	65.0	(56.4 – 72.8)	46.8	(41.5 – 52.2)	61.3	(55.7 – 66.5)	72.9	(67.7 – 77.6)
Multimorbidity (in previous 12 months)	74.9	(70.8 – 78.7)	67.8	(61.8 – 73.2)	70.9	(64.1 – 76.8)	69.5	(64.3 – 74.2)	63.5	(57.0 – 69.5)	62.2	(56.5 – 67.6)
Depressiveness^*^ (in previous 2 weeks)	16.6	(13.2 – 20.6)	15.0	(11.4 – 19.5)	13.1	(8.6 – 19.4)	14.2	(11.2 – 17.9)	11.6	(8.0 – 16.6)	3.9	(2.1 – 7.1)
(Very) high satisfaction with life^*^	75.8	(71.7 – 79.5)	74.8	(69.9 – 79.2)	81.9	(75.0 – 87.2)	79.8	(75.8 – 83.3)	77.5	(71.2 – 82.8)	90.5	(87.4 – 92.9)
Severely impaired vision	8.3	(6.5 – 10.5)	5.8	(4.0 – 8.2)	1.9	(1.1 – 3.5)	4.0	(2.8 – 5.8)	3.8	(2.3 – 6.2)	1.5	(0.8 – 2.8)
Severely impaired hearing	16.3	(13.6 – 19.4)	14.4	(11.1 – 18.6)	9.7	(6.1 – 15.1)	22.4	(19.1 – 26.1)	17.6	(13.5 – 22.7)	15.8	(12.0 – 20.5)
Impaired mobility	30.7	(26.8 – 35.0)	16.6	(13.2 – 20.7)	10.2	(6.8 – 14.9)	21.4	(17.7 – 25.8)	13.4	(9.9 – 17.8)	8.3	(5.8 – 11.7)
Chronic pain	19.0	(15.7 – 22.9)	17.2	(13.2 – 22.2)	7.5	(4.2 – 13.0)	12.8	(10.1 – 16.2)	8.2	(5.4 – 12.3)	7.7	(4.3 – 13.4)
Falls (in previous 12 months)												
≥ 1 fall	28.3	(24.8 – 32.1)	29.2	(24.7 – 34.2)	26.5	(20.8 – 33.3)	20.7	(17.8 – 24.0)	17.2	(12.6 – 22.9)	17.6	(14.4 – 21.4)
≥ 2 falls	15.8	(12.8 – 19.5)	14.6	(11.6 – 18.2)	9.5	(6.0 – 14.8)	12.5	(10.0 – 15.6)	8.7	(5.7 – 13.0)	9.1	(6.6 – 12.2)
Urinary incontinence (in previous 12 months)	33.1	(29.3 – 37.2)	28.2	(23.1 – 34.0)	28.2	(20.6 – 37.3)	26.9	(22.7 – 31.5)	19.6	(15.1 – 24.9)	18.3	(15.0 – 22.3)
Faecal incontinence (in previous 4 weeks)	13.9	(11.1 – 17.3)	9.6	(7.0 – 13.0)	9.1	(5.5 – 14.7)	7.4	(5.0 – 11.0)	5.9	(3.9 – 8.7)	4.5	(2.9 – 7.1)
Subjective deterioration of memory^*^	28.8	(24.5 – 33.4)	24.1	(19.9 – 28.8)	30.9	(24.8 – 37.8)	27.5	(23.7 – 31.7)	29.5	(23.7 – 36.0)	24.5	(20.5 – 29.1)

CI = confidence interval

^*^ = no proxy data included

^♦^ Classification Comparative Analyses of Social Mobility in Industrial Nations (CASMIN) Classification based on the highest level of education and training attained. A low education group was assigned if the respondent reported at most a lower secondary school-leaving certificate, but no vocational qualification

**Table 5:** Prevalences of health indicators by Region of residence (n = 3,694, weighted analyses) Source: Gesundheit 65+, own description

	Region of residence			
	West Germany (n = 2,980)		East Germany (n = 714)	
	%	(95 % Cl)	%	(95 % Cl)
**Living environment**				
Degree of care	16.6	(15.0 – 18.4)	18.0	(14.0 – 23.0)
Care provided to a person	12.0	(10.4 – 13.8)	11.1	(8.0 – 15.2)
Low social support	18.8	(16.6 – 21.2)	20.8	(16.8 – 25.5)
Loneliness^*^	19.0	(17.1 – 21.1)	19.7	(14.8 – 25.7)
**Activities/participation**				
Limitations in basic activities of daily life	9.5	(8.3 – 10.9)	10.9	(8.1 – 14.6)
Limitations in instrumental activities of daily life	20.7	(18.8 – 22.7)	19.2	(15.2 – 23.9)
**Health/functional resources**				
(Very) good subjective health	52.8	(50.2 – 55.3)	49.3	(43.6 – 54.9)
Multimorbidity (in previous 12 months)	68.5	(65.8 – 71.2)	71.6	(67.0 – 75.9)
Depressiveness^*^ (in previous 2 weeks)	13.1	(11.6 – 14.9)	14.9	(11.6 – 19.0)
(Very) high satisfaction with life^*^	78.7	(76.4 – 80.9)	77.8	(73.6 – 81.6)
Severely impaired vision	5.5	(4.7 – 6.6)	4.7	(3.1 – 6.8)
Severely impaired hearing	16.8	(15.3 – 18.5)	17.5	(14.0 – 21.8)
Impaired mobility	20.6	(18.5 – 22.9)	21.4	(17.6 – 25.9)
**Health/functional resources**				
Chronic pain	13.9	(12.2 – 15.7)	15.8	(12.1 – 20.3)
Falls (in previous 12 months)				
≥ 1 fall	24.1	(22.0 – 26.3)	24.2	(20.4 – 28.4)
≥ 2 falls	12.8	(11.1 – 14.7)	13.2	(10.3 – 16.8)
Urinary incontinence (in previous 12 months)	28.2	(25.8 – 30.8)	25.1	(20.9 – 29.9)
Faecal incontinence (in previous 4 weeks)	10.2	(8.7 – 12.0)	7.1	(5.3 – 9.5)
Subjective deterioration of memory^*^	26.8	(24.4 – 29.4)	28.9	(23.5 – 34.9)

CI = confidence interval

^*^ = no proxy information included
